# Utility of an Anterior Tibial Perforator for Skin Paddle Viability in Through-and-Through Defects of the Mandible

**Published:** 2018-09-20

**Authors:** Ivan E. Rodriguez, Becky B. Trinh, Frederic W-B Deleyiannis

**Affiliations:** Division of Plastic & Reconstructive Surgery, Department of Surgery, University of Colorado Anschutz Medical Campus; Aurora

**Keywords:** fibular free flap, mandibular reconstruction, anterior tibial artery, flow-through technique, de-epithelialization

## Abstract

**Objective:** The present report describes an alternative technique of using a flow- through, double-paddle, fibular, osteocutaneous free flap based on one perforator from the peroneal system and one perforator from the anterior tibial system for a through-and-through mandibular defect. **Methods:** The patient was a 65-year-old man who underwent a composite resection of the floor of the mouth, mandible, and chin pad due to recurrent oral cavity squamous cell carcinoma. The fibula was harvested with one posterior-lateral septal perforator from the peroneal system and with a second skin perforator from the anterior tibial system to perfuse a 15 × 14 cm skin island. The anterior tibial perforator was anastomosed to the distal end of the peroneal artery in a flow-through technique, and the area between the peroneal and tibial perforators was de-epithelialized to reconstruct separately the floor of mouth and cutaneous defects. **Results:** Good inflow and outflow of both skin islands were noted at the end of the procedure, and the patient recovered successfully without any fistulas or donor site morbidity. **Conclusions:** Perforators from the anterior tibial system should be considered for large, through-and-through mandibular defects when using 2 perforators from the peroneal system is not possible. In addition, we believe the flow-through technique can be useful in patients with vessel-depleted necks and provides a suitable match for vessel size between an anterior tibial perforator and the distal end of the peroneal system.

For mandibular reconstruction, fibular free flaps are regarded as the gold standard due to the reliable bone and skin that can be harvested.^1^ Large through-and-through defects present a challenge due to the need for 2 skin paddles. Three options are routinely used for through-and-through defects:

1. A fibular osteocutaneous flap with a wide skin paddle that is de-epithelialized and folded on itself.^2^^,^^3^
2. A fibular free flap with a second fasciocutaneous free flap.^4^^,^^5^
3. A fibular free flap with a pedicled flap (ie, pectoralis flap).^6^

If a de-epithelialized, fibular, osteocutaneous flap is used, one strategy to improve the blood supply to the 2 skin islands that are on either side of the area of de-epithelialization is to center one peroneal perforator in each skin island. However, this is often not possible since peroneal perforators originate along the length of the fibula and are not transversely separated. In this article, we present an alternative technique of using a flow-through, double-paddle, fibular, osteocutaneous free flap based on one perforator from the peroneal system and a second perforator from the anterior tibial system.

## CASE PRESENTATION

**Figure 1:**
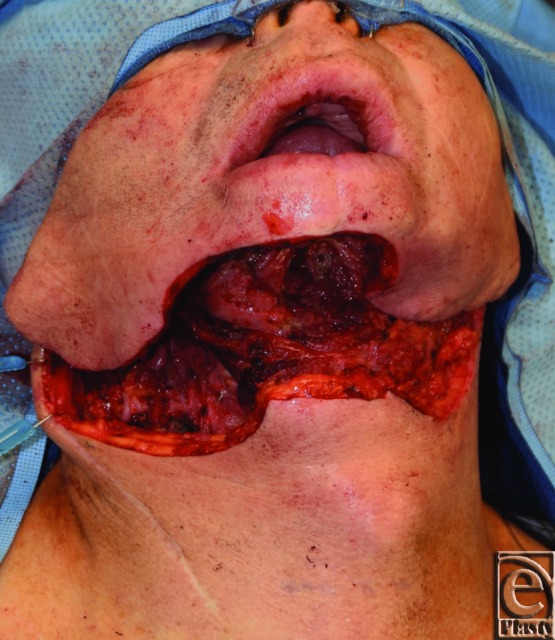
Composite oral cavity defect. Through-and-through defect following composite mandibular resection and resection of the entire chin pad.

**Figure 2:**
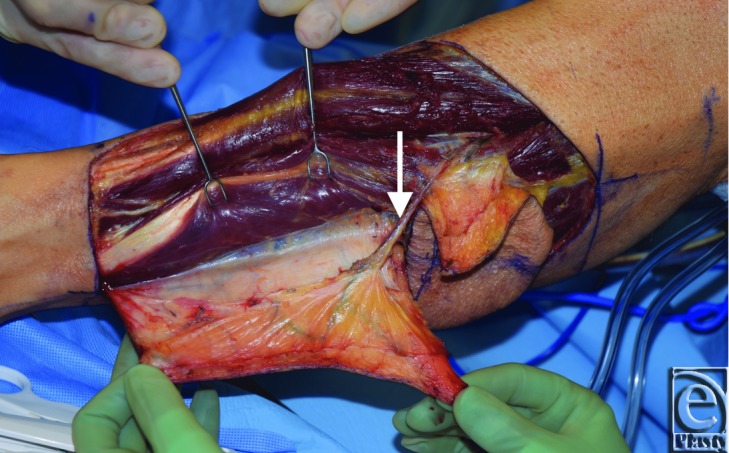
Simultaneous harvest of anterior tibial perforator free flap and fibular osteocutaneous free flap. Anterior tibial perforator supplying the anterior border of skin paddle.

**Figure 3:**
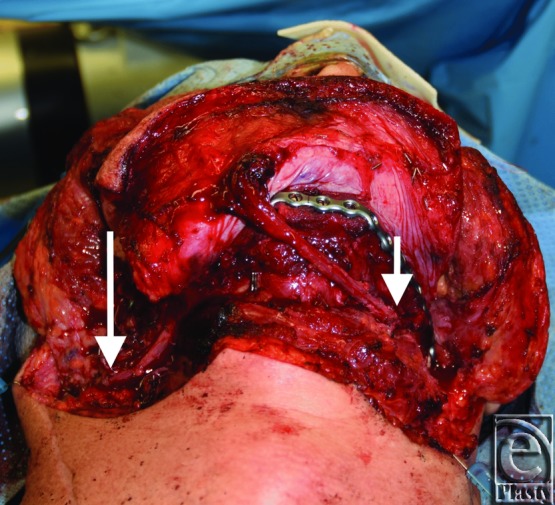
Fibular free flap plated into a reconstruction plate, shown with attached anterior tibial perforator flap (ie, flow-through free flap). Anterior tibial perforator sewn to the distal end of the peroneal artery, microvascular anastomosis for flow-through free flap performed in the leg (short arrow). Vasculature of fibula anastomosed to the right facial artery, the external jugular vein, and a large common facial vein (long arrow).

**Figure 4:**
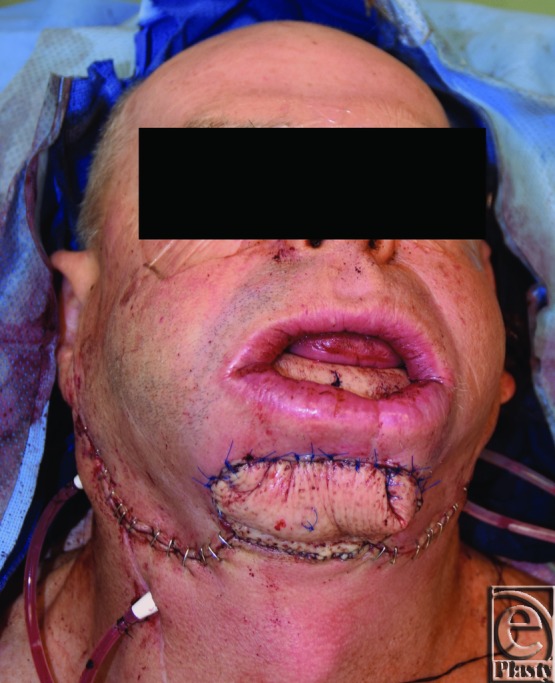
Inset of fibular flow-through free flap. Reconstruction of composite defect. Blue stitch located at the site of anterior tibial perforator.

The patient was a 65-year-old man with recurrent oral cavity squamous cell carcinoma who underwent a composite resection of the floor of mouth, the mandible from angle to angle, and the entire chin pad (Fig 1). The defect was addressed by designing a left fibular free flap with skin paddle 15 cm in length and 14 cm in width (ie, mid-calf: from the border of the lateral tibia to the mid-posterior line of the calf). Once an anterior incision was made, an anterior-lateral perforator supplying the skin paddle was encountered (Fig 2). This was skeletonized through the anterior compartment of the leg down to the anterior tibial artery and vein and prepared as a separate pedicle. The fibula was then harvested with one posterior-lateral septal perforator to the skin paddle. Four osteotomies were made in the fibula to reconstruct the angle-to-angle bony defect. The skin paddle was then draped over the bony reconstruction, with the area of de-epithelialization between the 2 perforators. The anastomoses of the peroneal artery and its 2 venae comitantes were done to the right facial artery, the right external jugular vein, and the right common facial vein, respectively. The pedicle to the anterior tibial perforator was then sewn to the distal ends of the peroneal artery and of one of the venae comitantes (ie, flow-through technique) (Fig 3). The peroneal perforator was centered in the skin paddle for the floor of mouth reconstruction, and the tibial perforator was centered in the skin paddle of the chin (Fig 4).

## DISCUSSION

When reconstructing a through-and-through composite mandibular defect with a de-epithelialized skin paddle from the fibula, the cutaneous defect should be at or below the plane of the mandible. By draping the skin paddle over the reconstructed mandible instead of insetting the skin paddle higher in the mid-cheek or lip, there is a reduced risk of an oral cutaneous fistula because gravity (and the septum attaching the skin paddle to the fibula) tends to pull the skin paddle back to the plane of the fibular inset. For defects that extend into the cheek or involve the majority of the vertical height of the lip, a separate fasciocutaneous free flap, in addition to a fibular osteocutaneous free flap, is recommended.^7^

If a separate perforator is not located in both the cutaneous and the oral cavity skin paddle, de-epithelialization can sometimes compromise the blood supply to one of the skin paddles. Often the skin paddle without the perforator will appear slightly blue (ie, venous congested) immediately after the de-epithelialization. In addition, it is often difficult to evert the skin edges at the site of the de-epithelialization, which can lead to the formation of oral cutaneous fistulas. One can score/cut below the dermis for better eversion, but this may jeopardize the subdermal blood supply to the skin paddle, which does not contain the perforator.

The use of a second pedicle from the anterior tibial system (ie, an additional skin perforator) improves arterial inflow and venous outflow. Given the independent blood supply, the skin paddles could also be completely divided to allow greater flexibility during the inset.

Septocutaneous perforators from the anterior tibial system routinely run in the anterolateral septum between the peroneal musculature and the anterior tibialis muscle or directly traverse the crural fascia at the anterior border of the tibia.^8^ In terms of anatomical distribution along the axis of the leg, Schaverien and Saint-Cyr^9^ identified 2 separate clusters of perforators greater than 0.5 mm in diameter, at 4 to 9 cm, and at 21 to 26 cm proximal to the intermalleolar line, containing 22% and 29% of all anterior tibial perforators, respectively. Hupkens et al^10^ identified the anterior tibial artery as the main supplier to the proximal and middle thirds of the leg, with 37% of all anterior tibial perforators concentrated in the proximal third and a mean pedicle length of 5.12 cm and a mean diameter of 1.04 mm.

## CONCLUSIONS

The novel surgical approach of harvesting a perforator from the anterior tibial system was chosen to provide a large, well-perfused skin paddle, while avoiding the added morbidity of a second donor site. We propose the use of this technique in large through-and-through mandible defects where using 2 perforators from the same peroneal system is not possible.

In addition, we believe the flow-through technique can be useful in patients with vessel-depleted necks and provides a suitable match for vessel size between an anterior tibial perforator and the distal end of the peroneal system.
